# Diversity of the Gut Microbiota in Dihydrotestosterone-Induced PCOS Rats and the Pharmacologic Effects of Diane-35, Probiotics, and Berberine

**DOI:** 10.3389/fmicb.2019.00175

**Published:** 2019-02-08

**Authors:** Feifei Zhang, Tong Ma, Peng Cui, Amin Tamadon, Shan He, Chuanbing Huo, Gulinazi Yierfulati, Xiaoqing Xu, Wei Hu, Xin Li, Linus R. Shao, Hongwei Guo, Yi Feng, Congjian Xu

**Affiliations:** ^1^School of Public Health, Fudan University, Shanghai, China; ^2^Obstetrics and Gynecology, Shanghai Medical College, Fudan University, Shanghai, China; ^3^Shanghai Key Laboratory of Female Reproductive Endocrine Related Diseases, Shanghai, China; ^4^Department of Integrative Medicine and Neurobiology, School of Basic Medical Sciences, Institutes of Brain Science, Brain Science Collaborative Innovation Center, State Key Laboratory of Medical Neurobiology, Fudan Institutes of Integrative Medicine, Fudan University, Shanghai, China; ^5^Department of Physiology/Endocrinology, Institute of Neuroscience and Physiology, The Sahlgrenska Academy, University of Gothenburg, Gothenburg, Sweden

**Keywords:** gut microbiota, PCOS, DHT, Diane-35, probiotics, berberine

## Abstract

Polycystic ovary syndrome (PCOS) is a frequent endocrine and metabolic syndrome in reproductive-age women. Recently, emerging evidence has shown that gut microbiota is closely related to metabolic diseases such as type 2 diabetes, obesity and PCOS. In the present study, we established dihydrotestosterone (DHT)-induced PCOS rats and used Illumina MiSeq sequencing (PE300) to examine the composition, diversity, and abundance of the gut microbiota in PCOS. We compared the effects of three PCOS treatments: Diane-35 (estrogen and progesterone), probiotics and berberine. The DHT-induced rats showed constant estrous cycles, the loss of mature ovarian follicles, insulin resistance and obesity. The reproductive and metabolic functions in the PCOS rats were improved by treatment with Diane-35 and probiotics. Diane-35 and probiotics could restore the diversity of the gut microbiota, and the recovery of gut microbiota disorders improved the reproductive function in PCOS-like rats. However, berberine drastically reduced the species diversity and amount of gut microbiota and showed no improvement in PCOS. The findings of this study will help us to better understand the influence of the gut microbiota in the metabolic and reproductive alterations in PCOS as well as suggest opportunities for future personal dietary guidance for PCOS.

## Introduction

Polycystic ovary syndrome (PCOS) is a common endocrine and metabolic syndrome among women of reproductive-age, with a worldwide incidence of 5–15% (Michelmore et al., 1999; March et al., 2010; Li R. et al., 2013). The principal characters are clinical or biochemical androgen excess, ovulation disorders and polycystic ovarian change (Conway et al., 2014; Skubleny et al., 2016), and it is associated with abdominal obesity, insulin resistance, impaired glucose metabolism, and dyslipidemia (Tan et al., 2010). The pathologic state of PCOS is a life-long condition, leading to an increased risk of hyperlipidemia, cardiovascular disease, hypertension, metabolic syndrome and endometrial cancer (Ali, 2015).

The pathogenesis of PCOS is still poorly understood, but the majority of researchers agree that it is a multifactorial disorder mainly induced by genetic and environmental factors. Even in lean PCOS patients, hepatic insulin resistance is also present (Conway et al., 2014). Compared to healthy lean and obese women, both lean and obese women with PCOS show a clustering of metabolic disorders, including high of total cholesterol, low-density lipoprotein cholesterol, triglycerides and low levels of high-density lipoprotein cholesterol levels (Diamanti-Kandarakis et al., 2007). Lifestyle modification, anti-androgenic agents, insulin-sensitizing agents, anti-hypertensives, and statins are all optional therapies in the clinic (Moghetti et al., 2000; Lord et al., 2003; Ibanez and de Zegher, 2004; Teede et al., 2010; Bargiota and Diamanti-Kandarakis, 2012), but caloric restriction and regular exercise are the primary recommendations (Morgante et al., 2018).

Evidences have shown that the increased risk of adiposity in PCOS compared with that in the general population (Hoeger and Oberfield, 2012). Changes in diet that increase the protein/carbohydrate ratio have been shown to have small metabolic and reproductive improvements in PCOS patients (Moran et al., 2003), while obese women with PCOS treated with a 1,000 kcal, low fat diet for 6–7 months showed body weight (BW) loss on the order of 5–10% and considerable reductions in the clinical manifestations of PCOS by restoring ovulation, increasing the pregnancy rate, and reducing levels of insulin and androgens (Kiddy et al., 1992). Another study found that modification of the gut microbiota is one means to treat obesity by regulating the metabolic system (DiBaise et al., 2008). Therefore, a new hypothesis was raised that the intestinal flora might be strongly associated with the occurrence and maintenance of PCOS symptoms. Compared with healthy women, the diversity of gut microbiota in patients with PCOS were reduced, and hyperandrogenism, total testosterone, hirsutism were inversely associated with alpha diversity (Torres et al., 2018). Exposure to hyperandrogenemia during fetal development might thus result in long-term changes in gut microbiota and subsequent changes in cardiac and metabolic function in the daughters of PCOS patients.

Prenatal androgen exposure can cause infant gut dysbiosis and altered abundance of bacteria that produce short-chain fatty acid metabolites, suggesting that androgen excess in immature fetuses could result in long-term alterations in gut microbiota and lead to increased risk of developing PCOS (Sherman et al., 2018).

Around 10^13^–10^14^ microorganisms populate the adult intestines (Ley et al., 2005, 2006), and the enteric nervous system has been referred to as “the second brain” (Mayer, 2011). The gut microbiota play a key role in regulating energy balance and are involved in the development and progression of obesity and metabolic diseases (Parekh et al., 2014). The diversity of the gut microbiota is related to multiple features of metabolic syndrome, and the gut microbiota contribute to endotoxemia and are involved in inflammation, glucose tolerance, and insulin secretion (Cani et al., 2007a,b). Some of these associations suggest that the gut microbiome might even be more important for some of these changes than the patient’s age, sex, and genetic background (Rial et al., 2016). Le Chatelier et al. (2013) have found that reduced diversity in the gut microbiota is related with adiposity, insulin resistance and dyslipidemia. Decreased intestinal flora diversity can also be seen in metabolic syndrome alone, which is associated with genetic variation of the apolipoprotein A5 gene. In addition, *Lactobacillus* has been found to be correlated with central adiposity, fasting blood sugar and negatively correlated with HDL-C levels (Lim et al., 2017).

In order to explore the role of the gut microbiota in PCOS, we used Illumina Miseq sequencing to study the composition and diversity of the gut microbiota in dihydrotestosterone (DHT)-induced PCOS rats in comparison with high-fat diet (HFD)-induced obese rats. At the same time, we also analyzed the correlation of the gut microbiota with circulating steroid levels and various metabolic parameters and evaluated the effects of three clinically relevant PCOS treatments – Diane-35, probiotics, and berberine.

## Materials and Methods

### Animals and Treatments

Female Wistar rats of 21 days were randomly divided into the following six groups: Control, HFD, DHT, DHT + Diane-35, DHT + Probiotics, and DHT + Berberine (*n* = 6 for all groups). All rats were placed in 12 h light/12 h dark, 22 ± 2°C constant temperature and 45–55% humidity, free to eat and drink. The Control, DHT, DHT + Diane-35, DHT + Probiotics, and DHT + Berberine groups were fed with standard chow, energy%: 10.3% from fat, 65.5% from carbohydrate and 24.2% from protein, 3.52kcal/g (Shanghai SLAC Laboratory Animals), while the HFD group was fed a high-fat chow, energy%: 60% from fat, 20% from carbohydrates and 20% from protein, 5.24 kcal/g (Research Diets, D12492).

This study was carried out in accordance with the local ethics committee of Shanghai Medical College, Fudan University, approved the experimental procedure and protocols (No. 20150119-019).

**FIGURE 1:**
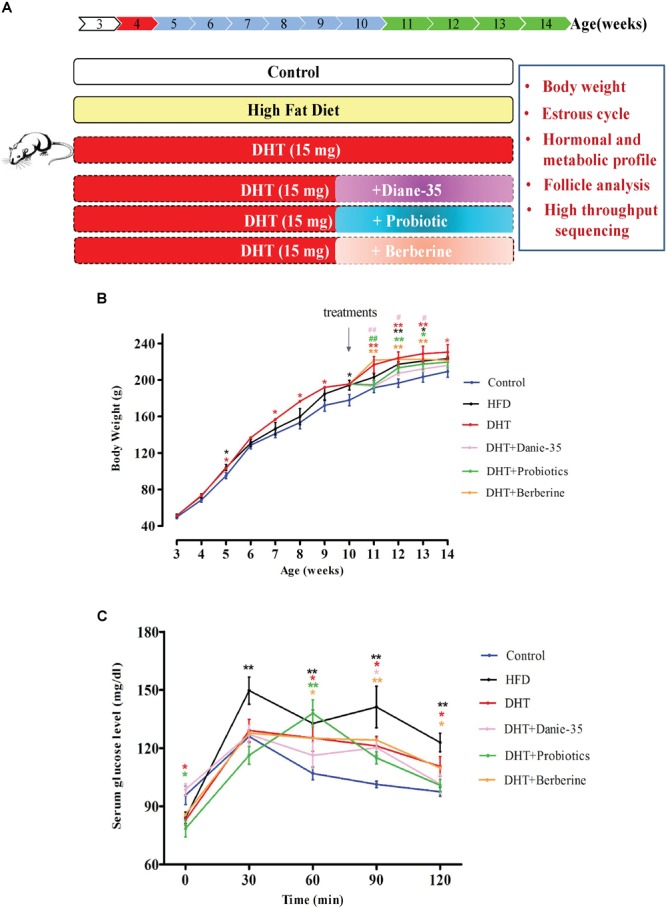
**(A)** Schematic of animals and treatments. Wistar rats were randomly divided into six group as shown. The control rats received blank cervical silicone tubes subcutaneously at 21 days of age. The HFD group was fed a diet in which 60% of the calories came from fat daily throughout the whole experiment. At the same time, silicone tubes with DHT (15 mg, slow releasing for 75 days) were implanted subcutaneously into the neck in the DHT, DHT + Diane-35, DHT + Probiotics, and DHT + Berberine groups. The DHT-induced PCOS rats (at 7 weeks after implantation) received Diane-35 (0.005 ml/kg BW), probiotics (*Bifidobacterium* triple viable, 210 mg/kg BW), or berberine (150 mg/kg BW) by intragastric administration. **(B)** Growth curves of rats from 21 days of age to 96 days of age. ^∗^*p* < 0.05, ^∗∗^*p* < 0.01 versus the control group and ^#^*p* < 0.05, ^##^*p* < 0.01 versus the DHT group using two-way ANOVA and Tukey’s *post hoc* test. **(C)** Oral glucose tolerance test (OGTT). The basal blood glucose level was measured before being given the oral D-glucose (1.5 g/kg BW), and then measurements were made at 30, 60, 90, and 120 min. ^∗^*p* < 0.05, ^∗∗^*p* < 0.01 versus controls.

The control rats received empty cervical silicone tubes subcutaneously (length = 1 cm, diameter = 2 mm) at 21 days of age (Figure 1A). At the same time, silicone tubes with DHT (15 mg, slow releasing for 75 days) were implanted subcutaneously into the neck in the DHT, DHT + Diane-35, DHT + Probiotics, and DHT + Berberine groups. At 7 weeks after implantation, the DHT-induced PCOS rats received Diane-35 [one tablet containing 2.0 mg cyproterone acetate and 35 μg ethinylestradiol dissolved in 50 ml 1% carboxymethyl cellulose (CMC) solution and administered at 0.005 ml/kg BW], probiotics Bifid Triple Viable (trade name Pei Feikang, a combination of *Bifidobacterium, Lactobacillus acidophilus*, and *Enterococcus faecali*, Shanghai Xinyi Inc., China) was dissolved in 1% CMC solution to a concentration of 42 mg/ml and administered at 210 mg/kg BW, or berberine (berberine hydrochloride tablets were dissolved in 1% CMC solution to a final concentration of 30 mg/ml and administered at 150 mg/kg BW), and all three were given by daily intragastric administration (Figure 1A, bottom panel). BW, vaginal opening, and estrus cycle were monitored throughout the experiment. An oral glucose tolerance test was performed at 8 a.m the day before the rats were sacrificed. At the termination of the experiment, all rats were deeply anesthetized to sacrificed, serum, bilateral ovaries, adipose tissue, muscles, liver, pancreas, and rectum fecal matter were collected. The control and HFD group were sacrificed at the diestrus stage of the estrous cycle.

#### Hormone Profile and Biochemical Indexes

Trunk circulation blood samples were obtained and were allowed to incubate for 4 h at room temperature. All samples were then centrifuged at 2,500 rpm for 15 min, collected into 1.5 ml Eppendorf tubes, and kept at -80°C for subsequent experiments. Progesterone, estradiol, total testosterone, follicle stimulating hormone (FSH), luteinizing hormone (LH), sex hormone-binding globulin (SHBG), and c-reactive protein (CRP) levels were measured using an enzyme-linked immunosorbent assay (ELISA) kit (Sino-UK Institute of Biological Technology, Beijing, China) with a STAT FAX 2100 Microplate Reader (Awareness Technology Inc., United States). TG, TC, HDL-C, LDL-C, insulin, aspartate transaminase (AST), and alanine aminotransferase (ALT) were measured using colorimetric kits (BioSino Bio-Technology & Science Inc., China) with a BS-420 Chemistry Analyzer (MINDRAY, China).

#### Vaginal Smears

The stage of the estrus cycle was determined by the predominant cell type in vaginal smears that were obtained from 7 weeks of age until the end of the experiment every day (Marcondes et al., 2002).

#### Oral Glucose Tolerance Test

After an overnight fasted (10–12 h), glucose levels in tail vein blood were measured with a glucometer (ACCU-CHECK Performa, Roche). One researcher held the animal securely and cleaned the tail, and a second researcher prepared the glucometer and took the measurements. Basal blood glucose levels were measured before administration of 50% oral D-glucose. After the measurement, the tail was covered with gauze, and the room temperature was kept constant throughout the process (2 g/kg BW), and measurements were then taken at 30, 60, 90, and 120 min.

### Microbial Diversity Analysis

#### Sample Collection

Fresh fecal samples were taken from the colons of all rats, collected into 1.5 ml sterile EP tubes, rapidly snap-frozen in liquid nitrogen, and stored at -80°C until further analysis.

#### DNA Extraction and PCR Amplification

Microbial DNA was extracted from the fecal samples with the E.Z.N.A.^®^ soil DNA Kit (Omega Bio-Tek, Norcross, GA, United States) according to the manufacturer’s protocol. The final DNA concentration and purity were determined with a NanoDrop 2000 UV-vis spectrophotometer (Thermo Scientific, Wilmington, NC, United States), and DNA quality was checked by 1% agarose gel electrophoresis. The V3-V4 hypervariable regions of the bacterial 16S rRNA gene were amplified with primers 338F (5′-barcode-ACTCCTACGGGAGGCAGCAG-3′) and 806R (5′-GGACTACHVGGGTWTCTAAT-3′) (Huang et al., 2016; Zheng et al., 2018) using a Thermocycler PCR system (GeneAmp 9700, ABI, Waltham, MA, United States). Each primer contained 8–13 bp paired-end error-correcting barcodes (Fadrosh et al., 2014). The barcodes were synthesized by Majorbio Bio-Pharm Technology Co. Ltd. (Shanghai, China). The PCR reactions were performed as follows: 3 min of denaturation at 95°C, 28 cycles of 30 s at 95°C, 30 s at 55°C, and 45 s at 72°C, and a final extension at 72°C for 10 min. The PCR reactions were performed in triplicate as 20 μL mixtures containing 4 μL 5× FastPfu Buffer, 2 μL 2.5 mM dNTPs, 0.8 μL each primer (5 μM), 0.4 μL of FastPfu Polymerase, 0.2 μL BSA, and 10 ng of template DNA. The resulting PCR products were separated on a 2% agarose gel and purified using the AxyPrep DNA Gel Extraction Kit (Axygen Biosciences, Union City, CA, United States) and quantified using QuantiFluor^TM^-ST (Promega, United States) according to the manufacturer’s protocol (Zheng et al., 2018). Sterile water was used as the negative control. The results of agarose gel electrophoresis of PCR products showed that the sterile water had no electrophoretic bands, indicating no contamination.

#### Library Preparation and Illumina MiSeq Sequencing

Library preparation involved four steps. (1) ‘Y’ adapters were linked. (2) Adapter dimers were removed by using beads. (3) The products were PCR amplified for library concentrations. (4) Single-stranded DNA fragments were generated using sodium hydroxide.

Sample libraries were pooled in equimolar amounts and paired-end (PE300, 2^∗^300 bp) sequenced on an Illumina MiSeq platform (Illumina, San Diego, CA, United States) according to the standard protocols^1^ in Majorbio Bio-Pharm Technology Co. Ltd. (Shanghai, China) (McElhoe et al., 2014). The raw reads were deposited into the NCBI Sequence Read Archive database (the accession number: SRP170100).

#### Processing of Sequencing Data

Raw fastq files were demultiplexed, quality-filtered by Trimmomatic, and merged by FLASH, and the reads were truncated where the average quality score was < 20 over a 50 bp sliding window. Sequences of each sample were separated according to the barcodes (exactly matching) and primers (allowing 2 nucleotide mismatching). We removed reads containing ambiguous bases, and we merged sequences with overlaps longer than 10 bp based on their overlap sequence.

We used UPARSE (version 7.1)^2^ with a 97% similarity cutoff to cluster operational taxonomic units (OTUs), and we used UCHIME to remove chimeric sequences. Finally, we used the RDP Classifier algorithm^3^ with a confidence threshold of 70% to analyze the taxonomy of each 16S rRNA gene sequence by comparing it against the Silva (SSU128) 16S rRNA database.

### Statistical Analysis

Statistics analysis was applied with SPSS 20.0 for Windows (SPSS Inc., Chicago, IL, United States).

All data were presented as mean ± SEM. One-way ANOVA followed by LSD test was used to determine the significance. *P* < 0.05 was set as statistically significant.

## Results

### Reproductive and Metabolic Disorders

Body weight was measured once a week. At 3 weeks of age, the BWs of all groups were not significantly different. After 2 weeks of DHT implantation, the BW of the DHT group was increased significantly compared to the controls (*p* < 0.05 or *p* < 0.01). The HFD group also had increased BW, but to a lesser degree than the DHT-induced rats. At 10 weeks of age, Diane-35, probiotics, and berberine were administered to the DHT-induced rats. Diane-35 and probiotics decreased the BW compared to the DHT group, but the effect of Diane-35 was the most obvious (Figure 1B).

The oral glucose tolerance test showed that circulating glucose levels were increased in the HFD group compared to the control group at 30, 60, 90, and 120 min after sugar taken and were higher in the DHT + Berberine group at 60, 90, and 120 min compared to the control group. Compared with the control group, glucose levels in the DHT + Diane-35 group were only higher at 90 min and were only higher at 60 min in the DHT + Probiotics group (Figure 1C).

**FIGURE 2:**
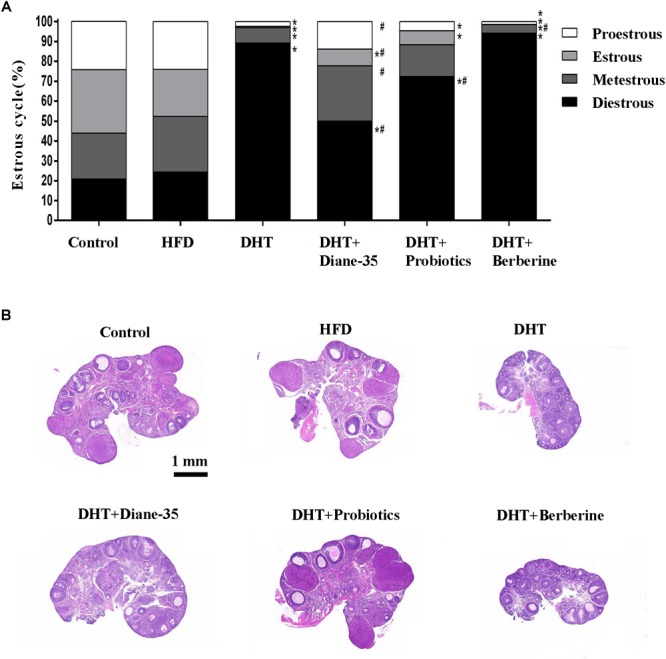
**(A)** The proportion of different estrous cycle stages in the six groups was analyzed using the Chi-square test, and differences were evaluated by *z*-test. ^∗^*p* < 0.05 vs. controls, ^#^ *p* < 0.05 vs. DHT. **(B)** H&E staining of the ovary in the control, HFD, DHT, and three treatment groups.

Ovarian function was evaluated based on estrous cyclicity, ovarian histology, and follicle number and morphology. As shown in Figure, the majority of the rats in the DHT and DHT + Berberine group were in the diestrus stage compared with the control and HFD group. Proestrus and estrus stages were observed to a greater degree in the DHT + Diane-35 and DHT + Probiotics group compared to the DHT group, but to a lesser degree compared to the control group (Figure 2A).

**FIGURE 3:**
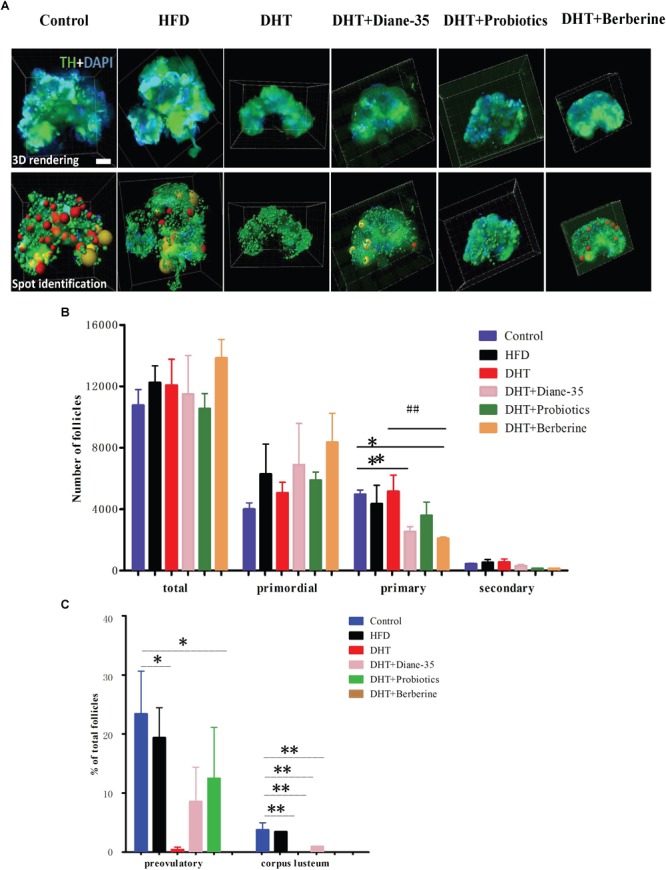
**(A)** 3D rendering after staining by TH and DAPI, with the bottom row showing the spot identification at the different follicle stages. Different stages of follicles are represented as different colors of spheres, of which the red and yellow represent preovulatory follicles and corpora lutea, respectively. **(B)** The numbers of total follicles and follicles at different stages in the six groups. **(C)** The percentages of different follicle stages in the six groups. TH, tyrosine hydroxylase; CL, corpus luteum. ^∗^*p* < 0.05, ^∗∗^*p* < 0.01 vs. control, ^##^*p* < 0.05 vs. DHT.

Morphological observation of the ovarian follicles showed that the HFD group had no differences in follicle number or composition compared with controls, but there were fewer mature follicles and corpus luteum in the DHT group compared to controls. The DHT + Diane-35 and DHT + Probiotics groups showed some corpus luteum and follicles at the antral and preovulatory stage, and follicular granulosa cells were arranged in an orderly fashion (Figure 2B). However, estrous cycle stage, ovarian morphology, and mature follicle number in the DHT + Berberine group showed no difference compared with the DHT group. The quantitation analysis of total follicles by Clarity 3D imaging showed that the number and the percentage of preovulatory follicles increased significantly in the DHT + Diane-35 and DHT + Probiotics groups compared with DHT, but the number of corpus luteum only increased in the DHT + Diane-35 group (Figure 3A–C).

The total testosterone concentrations were similar in DHT rats and controls, and progesterone decreased significantly in the DHT, DHT + Diane-35, DHT + Probiotics, and DHT + Berberine groups compared with the controls. The subcutaneous, visceral, and gonadal fat depot/BW ratios were higher in the DHT group compared to controls, and the subcutaneous fat depot/BW ratio was higher in the HFD group compared with the control group. The ovarian tissue/BW ratio was lower in the DHT, DHT + Berberine, DHT + Diane-35, and DHT + Probiotics groups compared with the control group. The uterine tissue/BW ratio was lower in the DHT group compared to controls (*p* < 0.05) (Supplementary Table 1).

### Diversity of the Gut Microbiota

V3 + V4 16S rRNA sequences reads with high quality and classification were obtained from the 36 samples with an average of 36,133 sequences per sample (the minimum of one sample was 30,242 reads and the maximum was 44,561 reads) (Supplementary Table 2).

**FIGURE 4:**
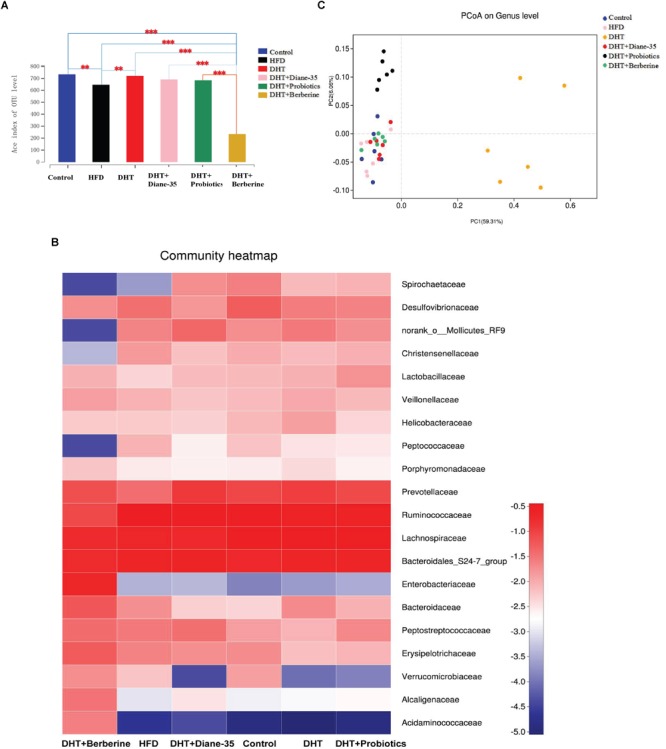
Community composition and alpha diversity of the gut microbiota in rats. **(A)** The ACE index shows the microbial diversity of each group. ^∗∗^*P* < 0.01, ^∗∗∗^*P* < 0.001 vs. Control. **(B)** The value in the heatmap represents the relative abundance of the top 20 families between groups. Each tile is colored by the log value of the relative abundance percentage. Each column in the heatmap represents a group, and each row represents a family-level taxon. **(C)** Principal coordinate analysis (PCoA) clustering according to similarity of gut microbiota composition in the samples. The DHT + Berberine group had the lowest diversity of gut microbiota, which clustered differently from the other groups.

In addition, we assessed the alpha diversity (ACE, Chao1, and Shannon and Simpson indices) in the samples. The Shannon–Wiener curve (smooth tendency) showed sufficient sample numbers. When the curve tended to be flat, it indicated that the amount of sequencing data was enough to reflect the vast majority of microbial diversity of the sample (Supplementary Figure 1). At a 97% similarity level, an average of 515 OTUs per sample were identified. The ACE, together with OTU, Chao1 coverage, and Shannon and Simpson analysis, showed the community diversity of all groups. Compared with the control, the DHT + Berberine group had significantly reduced alpha diversity of gut microbiota that clustered differently from the other two groups. The second lowest diversity was seen in the HFD group. Other groups showed no significant differences (Figure 4A and Supplementary Table 3). On the family level, the top 20 differentiated taxa with high relative abundance after the different treatments are shown in the heat-map. The community of the gut microbiota in the DHT + Berberine group was different from the other groups (Figure 4B), and unweighted-UniFrac-based principal coordinates analysis (PCoA) showed a distinct clustering of the microbiota composition in each group (*R*^2^= 0.78, *P* = 0.001, Adonis) (Figure 4C).

### The Composition of Gut Microbiota in Different Groups

**FIGURE 5:**
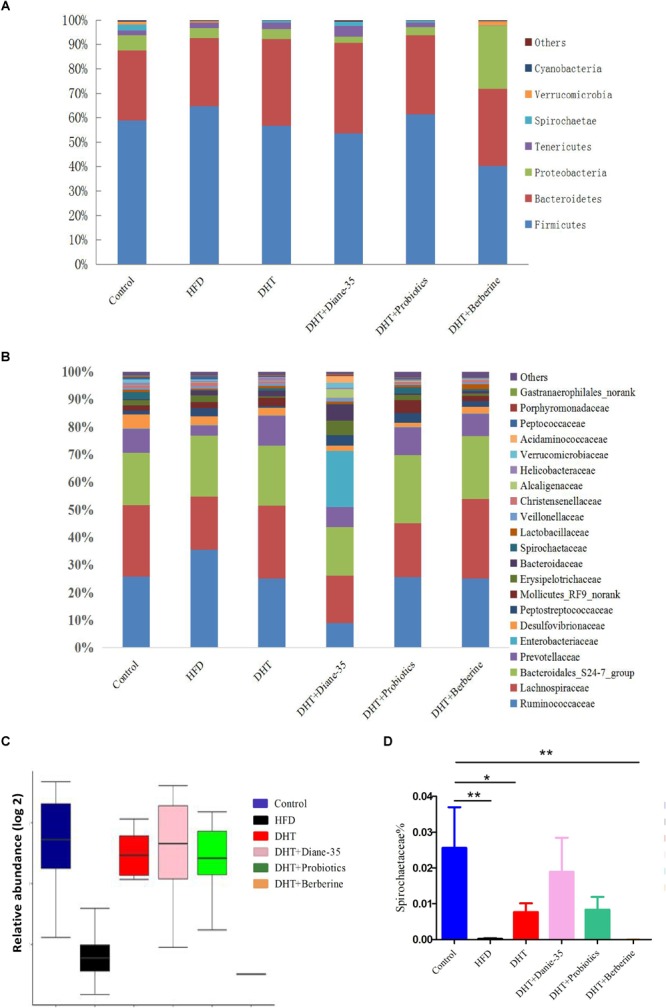
**(A)** Composition of the gut microbiota in different groups at the phylum level. **(B)** Composition of the gut microbiota in different groups at the family level. **(C)** The abundance of *Spirochaetae* at the phylum level. **(D)** The abundance of *Spirochaetaceae* at the family level. Values are presented as mean ± SEM, ^∗^*p* < 0.05, ^∗∗^*p* < 0.01.

*Firmicutes* and *Bacteroidetes* were the two dominant genera in the fecal samples from all groups, and the relative abundance of the top eight phyla is shown in Figure 5A. The ratio of *Firmicutes* to *Bacteroidetes* was 2.06 in controls, 2.32 in the HFD group, 1.60 in the DHT group, 1.27 in the DHT + Berberine group, 1.44 in the DHT + Diane-35 group, and 1.90 in the DHT + Probiotics group. Figure 5B shows the composition of the gut microbiota in different groups at the family level. The abundance of *Prevotellaceae* was 8.73% in controls, 3.66% in the HFD group, 10.96% in the DHT group, 8.27% in the DHT + Berberine group, 7.22% in the DHT + Diane-35 group, and 10.05% in the DHT + Probiotics group. The relative abundance of *Spirochaeta*is shown in Figure 5C. The abundance of *Spirochaetaceae*in controls (2.6%) and the DHT + Diane-35 group (1.8%) was significantly higher than that of the DHT (0.7%), DHT + Probiotics (0.8%), and DHT + Berberine (0.0007%) groups (Figure 5D).

**FIGURE 6:**
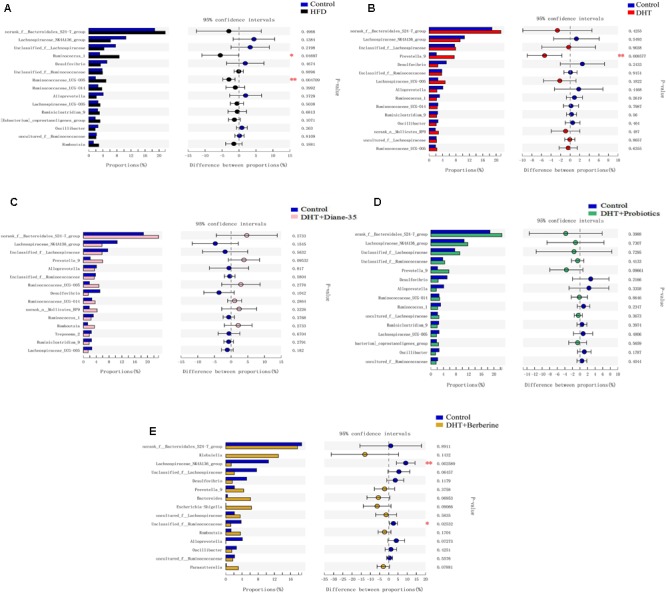
Different fecal microbiota in rats at the genus level. The histograms on the left represent the means of relative abundance of different microbiota in the group, and the right side shows the *p*-values and the 95% confidence intervals of the difference between groups. Statistical significance was defined as *p* < 0.05 using Student’s *t*-test. **(A)** The *Ruminococcus_1*, and *Ruminococcaceae_UCG-005* were significantly increased in the HFD group compared to control. **(B)** The *Prevotella_9* was decreased in the DHT group compared to control. **(C,D)** The different genera were similar between the control and the DHT + Diane-35 and DHT + Probiotics group. **(E)** *Lachnospiraceae_NK4A136_group, Unclassified_f__Ruminococcaceae*, were significantly decreased in the DHT + Berberine group compared to control group.

We used Student’s *t*-test to test for differences between groups and adjusted the *p*-values. Compared to control, the *Ruminococcus_1, Bacteroides, Prevotella_9, Trepo-nema_2*, and *Ruminococcaceae_UCG-005* were significantly increased in the HFD group (Figure 6A and Supplementary Figure 2A), while the genus *Prevotella_9, Bacteroides* was significantly increased and the genus *Prevotellaceae_Ga6A1_group*was decreased in the DHT group (Figure 6B and Supplementary Figure 2B). The different genera were similar between the controls and the DHT + Diane-35 and DHT + Probiotics groups (Figure 6C,D and Supplementary Figures 2C,D). In addition, *Lachnospiraceae_NK4A136_group, Unclassified_f_Ruminococcaceae, Ruminococcus_1, Ruminiclo-stridium_9, Lachnospiraceae_UCG-005, Ruminococcaceae_UCG-014, and _UCG-005*were significantly decreased in the DHT + Berberine group (Figure 6E and Supplementary Figure 2E).

### Correlation Between Gut Microbiota and Clinical Variables

**FIGURE 7:**
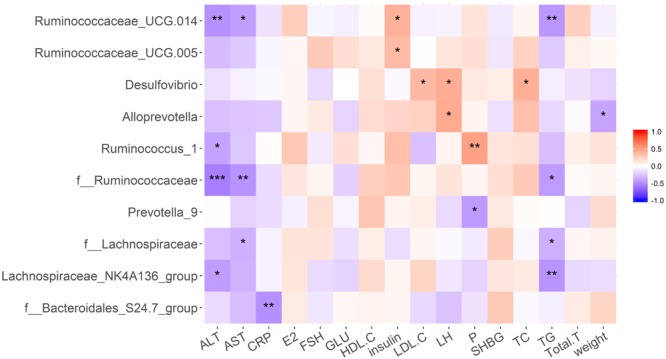
Pearson correlation analysis was performed to determine the correlation between clinical phenotypes and gut microbiota in each group. ^∗^*p* < 0.05, ^∗∗^*p* < 0.01, ^∗∗∗^*p* < 0.001.

Correlation analyses were performed to determine the potential associations between microbial genera and metabolic disorders changes (Figure 7). Microbes that maintain the intestinal micro-ecological balance such as *Bacteroidales-S24-7* showed a negative relationship with CRP. *Lachnospiraceae NK4A136* showed a negative relationship with AST and TG. *Ruminococcus_1* showed a negative relationship with ALT, but a positive relationship with progesterone. Candidate pathogens have opposite relationship with some clinical variables. For example, *Prevotella_9* was negative correlation with progesterone, and all of the *Desulfovibrio* displayed a positive correlation with TC and LH.

## Discussion

In addition to reproductive disorders, the DHT-induced PCOS model rats in this study showed many metabolic syndromes similar to women with PCOS, including increased BW, abnormal glycolipid metabolism, and intestinal flora disturbance. We found that the abundance and diversity of intestinal flora decreased significantly in the HFD group due to the high fat diet and in the DHT + Berberine group likely because of the strong antibacterial action of berberine. Smaller differences were seen among other groups. However, at the phylum level of gut flora, there was a consistent composition in the HFD and DHT groups, such as lower diversification of *Proteobacteria, Spirochaetae*, and *Verrucomicrobia*, and more diversified *Tenericutes.* The *Bacteroidetes* were increased in the DHT group. At family level, the abundances of the bacterial taxa in the HFD group were obviously different compared to the control group. In contrast, the DHT group had more similar abundances of different taxa compared to the controls except for *Bacteroidates_S24-7_group, Prevotellaceae, Enterobacteriaceae*, and *Desulfovibrionaceae*. Of the three treatments, Diane-35 and probiotics could restore the diversity and abundance of the gut flora, showing significant effects on *Firmicutes, Tenericutes, Proteobacteria*, and *Spirochaetae*. However, berberine significantly reduced the species diversity and number of intestinal flora and had no obvious effect on improving the symptoms of PCOS.

Long-term HFDs have been shown to result in the development of obesity-related metabolic disorders and concurrent diseases due to changes in the gut microbiota. HFDs decrease the diversity of the gut microbiota and alter bacteria composition, showing a decrease in *Bacteroidetes* and an increase in *Firmicutes* and *Protebacteria* or changes in the *Firmicutes* to *Bacteroidetes* ratio (Hildebrandt et al., 2009; Le Chatelier et al., 2013). A previous study showed that if the gut microbiota of an obesity-prone individual was transferred into bacteria-free mice, the latter gained BW and became obese, and in that work *Phascolarctobacterium, Proteus mirabilis*, and *Veillonellaceae* were positively correlated with all metabolic parameters, but only *Lactobacillus intestinalis* was negatively correlated with BW and fat mass (Lecomte et al., 2015). Regarding PCOS-related obesity, lower alpha diversity has been observed in women with PCOS and to be correlated negatively with hyperandrogenism, total testosterone, and the other primary reproductive and metabolic characteristics of PCOS (Torres et al., 2018). It was also reported that letrozole-induced PCOS rats show lower Lactobacillus, Ruminococcaceae, and Clostridium and higher Prevotellaceae (Guo et al., 2016), which is consistent with the results of our DHT-induced PCOS rats. Therefore, although the causes of obesity are different, HFD-induced and DHT-induced changes in intestinal flora are very similar.

Studies have shown that many genes related to the etiology of PCOS are involved in steroid synthesis and carbohydrate metabolism, suggesting that metabolic factors are involved in the development of PCOS (Ben-Shlomo, 2003; Fernandez-Real and Pickup, 2012; Shi et al., 2012). Research has also shown an increase of serum levels of zonulin, which is a biomarker for gut permeability in PCOS patients. Insulin resistance and menstrual disorders also demonstrate relevance with serum levels of zonulin, suggesting that changes in gut permeability might be involved in the pathophysiology of PCOS (Zhang et al., 2015). Sun analyzed the metabolic profile differences between PCOS patients and healthy women and showed that plasma levels of dimethylamine were significantly increased in PCOS patients, demonstrating that gut microflora was more active in the patients with PCOS.(Sun et al., 2012). Tremellen and Pearce (2012) suggested that dysbiosis of gut microbiota (the DOGMA theory) which induced by a high-fat, high-sugar diet, therefore contributes to higher intestinal permeability in women with PCOS. Lipopolysaccharides produced by gram-negative bacteria could then transfer through gut wall into the blood and caused a long term, low level inflammation. Subsequent immune system disrupted insulin receptors and derived up insulin levels, finally boosts testosterone produced by the ovaries causing PCOS. Thus the DOGMA theory describes how the gut microbiota might be directly related to the pathogenesis of PCOS (Tremellen and Pearce, 2012). Changes in gut microbiota have also been seen in letrozole-induced mouse models of PCOS (Conway et al., 2014; Guo et al., 2016), and fecal microbiota transplantation (FMT) from the PCOS-like mice to normal mice showed that the gut microbiota is also closely related with the host’s level of sex hormone, estrous cycles, and ovarian morphology (Guo et al., 2016). Steroids have also been shown to regulate the composition of the gut microbiome and metabolism and that “dysbiosis” in the gut microbiome might also occur in PCOS patients (Kelley et al., 2016).

We observed significant differences in *Spirochaetaceae* between the control group and the other groups except for the DHT + Diane-35 group, and a plausible mechanism might be that this species plays a role in insulin resistance. However, there is no research to prove that the *Spirochaetaceae* have a regulatory role in the intestinal flora, and this theory requires further experimental work. We also found that *Prevotella-9* was significantly increased in the DHT group, and *Prevotella* species have been found to be prevalent in the respiratory system, oral cavity, and genital tract and to contribute to infections such as chronic sinusitis, periodontitis, and bacterial vaginosis (Brook, 2005; Oakley et al., 2008; Teles et al., 2012). Males appear to be more likely to carry *Prevotella intermedia* in the oral cavity compared to females, suggesting that there is an association between sex hormones and *Prevotella* (Umeda et al., 1998). Among women with PCOS, the level of *Prevotella intermedia* serum antibodies were higher than in healthy women (Akcali et al., 2014), and PCOS rats treated with FMT and *Lactobacillus* transplantation had decreased prevalence of *Prevotella* and decreased levels of testosterone and androstenedione (Kelley et al., 2016).

Diane-35 is a common medication to treat PCOS, which composed of 2 mg cyproterone acetate and 0.035 mg ethinyl estradiol each tablet. Cyproterone acetate is not only the progesterone agonist, but also androgen antagonist. In addition, ethinyl estradiol might suppress ovarian estradiol synthesis through regulation of the hypothalamic-pituitary-ovarian axis. In the present study, Diane-35 not only restored the reproductive function significantly, but also increased the diversity and abundance of the gut microflora compared with the DHT group, showing a significant effect on the genus *Prevotella*. The abundance of *Eubacterium_ventriosum_group, Prevotellaceae_Ga6A1_group, Adlercreutzia*, and *Turicibacter* were significantly greater in the DHT + Diane-35 group compared to the DHT group, but these were all similar to the control group. Recent research has shown that prenatal androgen exposure in rats causes gut microbiota dysbiosis (Sherman et al., 2018), mainly in terms of changes in bacterial species that are involved in the short-chain fatty acids. The gut bacteria have been suggested to be another source of male steroids (Ridlon et al., 2013), and many bacterial species are known to convert glucocorticoids into androgens. Taken together, all of the evidence above indicates that there is a close interaction between androgens and the gut microbiota, and that this combined with hereditary and environmental factors is a primary risk factor for developing PCOS.

Probiotics have a long history and are a widely used food supplement to improve intestinal health, but only the strains classified as lactic acid bacteria are significant nutritionally, and the genera *Lactococcus* and *Bifidobacterium* are the most important (Holzapfel et al., 2001). Bifid Triple Viable (commercial name: Pei Feikang), which is a combination of *Bifidobacterium, Lactobacillus acidophilus*, and *Enterococcus faecali*, has been used in patients with diarrhea and ulcerative colitis (Li et al., 2008, 2012; Zhang et al., 2012), and these probiotics increased the diversity and added *Lactobacillus* in the DHT + Probiotics group compared with the DHT group, and this leads to dramatic decrease in the serum levels of cholesterol and increase in the serum levels of progesterone. In letrozole-induced PCOS rats, *Lactobacillus*-treated groups with increased numbers of *Lactobacillus* and *Clostridium* and decreased numbers of *Prevotella* had decreased androgen biosynthesis and normalized ovarian morphologies (Guo et al., 2016). These results indicated that probiotics are beneficial to the treatment of PCOS, and the possible mechanism is consistent with the DOGMA theory, including maintenance of the gut microbiota, improvement in intestinal permeability, and prevention of bacterial translocation from the gut.

Berberine is a quaternary ammonium salt derived from the protoberberine group of benzylisoquinoline alkaloids, extracted from the roots, rhizomes, stems, and bark of *Berberis, Coptis chinensis, Phellodendri chinensis*, etc. It has strong anti-bacterial, anti-lipid peroxidation, and anti-tumor activities, and it is under investigation to determine whether it might have applications for treating arrhythmia, diabetes, hyperlipidemia, and PCOS (Li Y. et al., 2013). Li recruited 120 patients with PCOS who were treated for 12 weeks with berberine or placebo, and berberine significantly improved insulin resistance in the PCOS patients (Li Y. et al., 2013). Another clinical trial also confirmed that 3 months of berberine treatment significantly decreased waist circumference and waist-to-hip ratio and reduced metabolic parameters compared with metformin and placebo (Wei et al., 2012). However, in our PCOS-like rats 4 weeks of berberine administration clearly decreased the composition and diversity of the gut microbiota and gave no improvement of any metabolic or reproductive phenotypes.

Dietary changes are considered the first line of treatment for those with PCOS (Moran et al., 2011). However, little is known about how often clinicians recommend this treatment, and there is currently no standard dietary approach recommended as optimal for such women (Moran et al., 2009). An ideal dietary plan recommended for PCOS patients should be composed of average amounts of polyunsaturated fatty acids, omega-3, and sufficient intake of fiber-rich foods such as whole grains, legumes, vegetables, and fruits with hypoglycemic index (Faghfoori et al., 2017).

PCOS and its progress to metabolic syndromes, type 2 diabetes mellitus, have traditionally been considered with intake of excess caloric a decrease of physical activity, and certain genetic factors. However, increasing evidence suggests that the intestinal microbiota are closely associated with these diseases (Turnbaugh et al., 2009; Theriot et al., 2014; Paramsothy et al., 2017).

FMT has been shown in animal models to be a potential treatment for improving obesity and its associated comorbidities, but there is little evidence regarding the use of FMT in treating obesity and metabolic syndrome in humans (Marotz and Zarrinpar, 2016). There are only two registered clinical trials using FMT as a treatment for obesity (clinicaltrials.gov identifiers: NCT02530385 and NCT02741518), and one for treating metabolic syndrome by FMT with donor fecal matter of lean people (Vrieze et al., 2012). The data from these trials and animal models suggest that FMT is a promising treatment for metabolic syndrome.

The associations between the gut microbiota and clinical variables presented here suggest that certain groups of microbes likely play important roles in the development of obesity-related metabolic disorders, and our results show that these bacteria might be of help for the prognosis, prevention, and treatment of PCOS. The findings of this study enable us to develop better understanding the influence of the gut microbiota in the metabolic and reproductive alterations in PCOS, and they also suggest opportunities for future personal dietary guidance, new biomarkers, and new treatments with specific groups of bacteria, and they support replacement with pathogen-free gut microbiota as a potential treatment for PCOS.

## Author Contributions

FZ, HG, YF, and CX conceived the experiments, designed the project and protocols, and developed the collaborations. FZ, TM, PC, SH, CH, GY, WH, XX, AT, and YF performed the experiments and analyzed the data. FZ, AT, CX, and YF wrote the manuscript. FZ, AT, LS, XL, HG, and CX provided scientific oversight and guidance and edited the manuscript. FZ, PC, TM, HG, AT, YF, and CX are the guarantors of this work and, as such, had full access to all of the data in the study and take responsibility for the integrity of the data and the accuracy of the data analysis.

## Conflict of Interest Statement

The authors declare that the research was conducted in the absence of any commercial or financial relationships that could be construed as a potential conflict of interest.

## Footnotes

1 https://support.illumina.com/downloads/miseq_system_user_guide_15027617.html

2 http://drive5.com/uparse/

3 http://rdp.cme.msu.edu/
