# Supplementary material for: Diversity of the Gut Microbiota in Dihydrotestosterone-Induced PCOS Rats and the Pharmacologic Effects of Diane-35, Probiotics, and Berberine
Source: Front Microbiol. 2019 Feb 8;10:175. doi: 10.3389/fmicb.2019.00175 (PMC6375883; doi:10.3389/fmicb.2019.00175)
Supplement: Supplementary file 1 [file Table_1.DOCX]

Supplementary Material

## 1.Supplemental Figures(PPT)

**Supplemental Figure 1.**The species Shannon curve

**Supplemental Figure 1.**The species Shannon curve (smooth tendency) shows the sufficient sample number of observed species. When the curve tends to be flat, this indicates that the amount of sequencing data is large enough to reflect the vast majority of the microbial diversity information in the sample.

**Supplemental Figure 2.Different fecal microbiota in rats at the genus level**

Supplementary Figure 2(A-E). Different fecal microbiota in rats at the genus level of relative abundance in top 30. The histograms on the left represent the means of relative abundance of different microbiota in the group, and the right side shows the p-values and the 95% confidence intervals of the difference between groups. Statistical significance was defined as p < 0.05 using Student’s t-test.

**Supplemental Tables**

**Supplemental Table 1:**Characteristics of endocrine, metabolism, and liver function alterations in DHT-induced PCOS-like rodent models.

| **Group (n = 6)** | **Control** | **HFD** | **DHT** | **Diane-35** | **Probiotics** | **Berberine** |
| --- | --- | --- | --- | --- | --- | --- |
| **Gonadotropins** |  |  |  |  |  |  |
| FSH (mIU/ml) | 3.87 ± 2.49 | 3.78 ± 1.87 | 2.66 ± 1.22 | 14.02 ± 11.73^*,##^ | 10.18 ± 8.91^*^ | 5.01 ± 6.01 |
| LH (mIU/ml) | 26.30 ± 10.04^##^ | 13.79 ± 2.73^**^ | 14.18 ± 6.20^**^ | 16.02 ± 6.41^*^ | 11.97 ± 7.84^**^ | 12.13 ± 8.29^**^ |
| **Steroid hormones** |  |  |  |  |  |  |
| E2（pg/ml） | 125.25 ± 26.1 | 73.69 ± 63.94^**,#^ | 115.13 ± 23.25 | 16.14 ± 3.86^**,##^ | 14.61 ± 5.54^**,##^ | 23.47 ± 6.91^**,##^ |
| T（ng/ml） | 0.3 ± 0.89 | 0.36 ± 0.11 | 0.28 ± 0.16 | 0.26 ± 0.00 | 0.34 ± 0.18 | 0.32 ± 0.35 |
| P（ng/ml） | 0.44 ± 0.12^##^ | 0.46 ± 0.94^##^ | 0.18 ± 0.06^**^ | 0.29 ± 0.17^*^ | 0.24 ± 0.11^**^ | 0.29 ± 0.08^**^ |
| SHBG(nmol/L) | 72.86 ± 6.14 | 74.71 ± 8.83 | 77.47 ± 5.74 | 67.98 ± 4.72^*^ | 74.14 ± 5.47 | 70.49 ± 2.95^*^ |
| INS (uIU/ml) | 29.27 ± 3.10 | 33.76 ± 7.02 | 28.76 ± 7.49 | 33.43 ± 7.20 | 27.58 ± 6.69 | 22.83 ± 4.78 |
| **Lipid profile** |  |  |  |  |  |  |
| TC (mmol/L） | 2.33 ± 0.28^##^ | 2.08 ± 0.23 | 1.80 ± 0.34^**^ | 2.13 ± 0.30 | 1.87 ± 0.25^**^ | 1.77 ± 0.42^**^ |
| TG (mmol/L） | 0.51 ± 0.19 | 0.53 ± 0.11 | 0.50 ± 0.03 | 0.65 ± 0.18 | 0.50 ± 0.12 | 0.94 ± 0.27^**,##^ |
| HDL –C (mmol/L） | 0.79 ± 0.07 | 0.66 ± 0.08 | 0.69 ± 0.15 | 0.82 ± 0.21 | 0.71 ± 0.09 | 0.62 ± 0.16^**^ |
| LDL-C (mmol/L） | 0.74 ± 0.15^#^ | 0.52 ± 0.22^**^ | 0.56 ± 0.09^*^ | 0.64 ± 0.09 | 0.60 ± 0.09 | 0.57 ± 0.10^**^ |
| **Liver function** |  |  |  |  |  |  |
| AST (U/L) | 64.83 ± 20.24 | 71.42 ± 30.30 | 82.27 ± 14.48 | 77.98 ± 27.37 | 86.73 ± 19.12 | 153.67 ± 78.13^**,##^ |
| ALT (U/L) | 22.91 ± 18.71 | 15.22 ± 2.89 | 26.15 ± 4.53 | 26.64 ± 7.38 | 24.46 ± 3.37 | 51.07 ± 26.56^**,##^ |
| CRP (mg/L) | 3.23 ± 1.18 | 5.30 ± 1.19^*^ | 4.57 ± 1.57 | 4.51 ± 0.89 | 5.16 ± 1.88^*^ | 5.71 ± 2.53^**^ |
| **Fat depots/BW ratio** |  |  |  |  |  |  |
| Subcutaneous (g/kg BW) | 1.87 ± 0.46^#^ | 3.21 ± 0.73^*^ | 3.04 ± 1.17* | 2.36 ± 0.21 | 1.67 ± 0.84^##^ | 2.11 ± 0.71 |
| Visceral (g/kg BW) | 6.13 ± 1.04 | 10.89 ± 3.64^**,##^ | 5.53 ± 2.59 | 5.62 ± 0.62 | 5.43 ± 0.96 | 5.23 ± 1.73 |
| Gonadal (g/kg BW) | 8.58 ± 3.39 | 14.68 ± 1.63^**,##^ | 9.63 ± 2.56 | 7.92 ± 2.10 | 8.91 ± 1.78 | 10.84 ± 2.58* |
| **Tissues/BW ratio** |  |  |  |  |  |  |
| Tibialis anterior muscle  (g/kg BW) | 1.74 ± 0.24 | 1.65 ± 0.11 | 1.68 ± 0.58 | 1.91 ± 0.23 | 1.74 ± 0.23 | 1.65 ± 0.25 |
| Soleus muscle  (g/kg BW) | 4.79 ± 0.45 | 4.21 ± 0.21 | 4.31 ± 0.77 | 4.86 ± 0.35 | 5.43 ± 0.85 | 3.88 ± 1.79 |
| Extensor digitorum longus (g/kg BW) | 0.56 ± 0.20^#^ | 0.41 ± 0.01^*^ | 0.40 ± 0.04^*^ | 0.38 ± 0.07^*^ | 0.39 ± 0.09^*^ | 0.45 ± 0.11 |
| Uterus (both) (g/kg BW) | 1.70 ± 0.50^#^ | 2.02 ± 0.96^##^ | 0.84 ± 0.61^*^ | 1.22 ± 037 | 1.16 ± 0.66 | 1.70 ± 0.38^#^ |
| Ovary (both) (g/kg BW) | 0.21 ± 0.04^##^ | 0.20 ± 0.03^##^ | 0.07 ± 0.03^**^ | 0.12 ± 0.08^*^ | 0.13 ± 0.08^*^ | 0.08 ± 0.01^**^ |
| Liver (g/kg BW) | 26.03 ± 3.21 | 26.40 ± 1.81 | 26.13 ± 2.93 | 29.00 ± 3.16^*^ | 27.94 ± 1.39 | 29.72 ± 4.21 |
| Pancreas (g/kg BW) | 5.82 ± 1.47 | 4.70 ± 0.33 | 4.84 ± 0.68 | 5.00 ± 0.68^*^ | 5.11 ± 0.69 | 4.67 ± 1.09* |

Results are presented as the mean ± SEM, A p-value less than 0.05 was considered statistically significant. * p < 0.05 versus the Control group, ** p < 0.01 versus the Control group, ^#^p < 0.05 versus the DHT group, ^##^ p < 0.01 versus the DHT group.

**Supplemental Table 2.** The number of sequences of all samples.

| Group | Sequences**(Average)** | Sequences **(min)** | Sequences **(max)** |
| --- | --- | --- | --- |
| **Control** | 35,505 | 30,330 | 40,783 |
| **HFD** | 36,044 | 33,618 | 42,321 |
| **DHT** | 39,133 | 32,945 | 44,561 |
| **DHT+Diane-35** | 31,907 | 30,242 | 33,636 |
| **DHT+Probiotics** | 38,470 | 32,367 | 43,197 |
| **DHT+Berberine** | 38,824 | 30,630 | 44,014 |

**Supplemental Table 3.** The alpha diversity analysis of the different groups (mean ± SEM).

| **Group** | **OTUs**^1^ | **Shannon**^2^ | **Simpson**^3^ | **Chao1**^4^ | **ACE**^5^ | **Good coverage**^6^ |
| --- | --- | --- | --- | --- | --- | --- |
| **Control** | 626 ± 41 | 4.94 ± 0.28 | 0.017 ± 0.009 | 713 ± 27 | 712 ± 39 | 0.995 ± 0.000 |
| **HFD** | 528 ± 31** | 4.63 ± 0.19 | 0.025 ± 0.005 | 622 ± 33** | 616 ± 29** | 0.995 ± 0.000 |
| **DHT** | 597 ± 24 | 4.91 ± 0.11 | 0.019 ± 0.004 | 712 ± 33 | 691 ± 23 | 0.995 ± 0.000 |
| **DHT+Danie-35** | 600 ± 31 | 4.79 ± 0.26 | 0.025 ± 0.010 | 692 ± 34 | 683 ± 31 | 0.995 ± 0.001 |
| **DHT+Probioticss** | 573 ± 47 | 4.82 ± 0.21 | 0.022 ± 0.007 | 662 ± 49 | 651 ± 50 | 0.995 ± 0.000 |
| **DHT+Berberine** | 187 ± 29** | 3.22 ± 0.56 ** | 0.095 ± 0.073** | 216 ± 37 ** | 218 ± 35 ** | 0.998 ± 0.001 ** |

**p < 0.01 versus control group

^1^The operational taxonomic units (OTUs) were defined with 3% dissimilarity level.

^2^ The Shannon index, which is a function of both the number of OTUs and their proportions, is maximized when each OTU has the same proportion, and it gets smaller as the variability in proportions increases. It was calculated with the formula E = H/ln(S), where H is the Shannon diversity index and S is the total number of sequences in that group .

^3^ The Simpson index is another estimator of alpha diversity. The larger the value, the lower the diversity.

^4^The Chao1 index was calculated by the Chao1 algorithm to estimate the number of OTUs. The larger the value, the greater the number of species in the samples.

^5^The abundance-based coverage estimator (ACE) was calculated to estimate the richness of the samples.

^6^ The Good Coverage, which is calculated by Good’s method using the MOTHUR program, was used to estimate the percentage of total species sequenced in the samples.
