# Supplementary material for: Diversity of the Gut Microbiota in Dihydrotestosterone-Induced PCOS Rats and the Pharmacologic Effects of Diane-35, Probiotics, and Berberine
Source: Front Microbiol. 2019 Feb 8;10:175. doi: 10.3389/fmicb.2019.00175 (PMC6375883; doi:10.3389/fmicb.2019.00175)
Supplement: Supplementary file 2 [file Presentation_1.PPTX]

## Slide 1
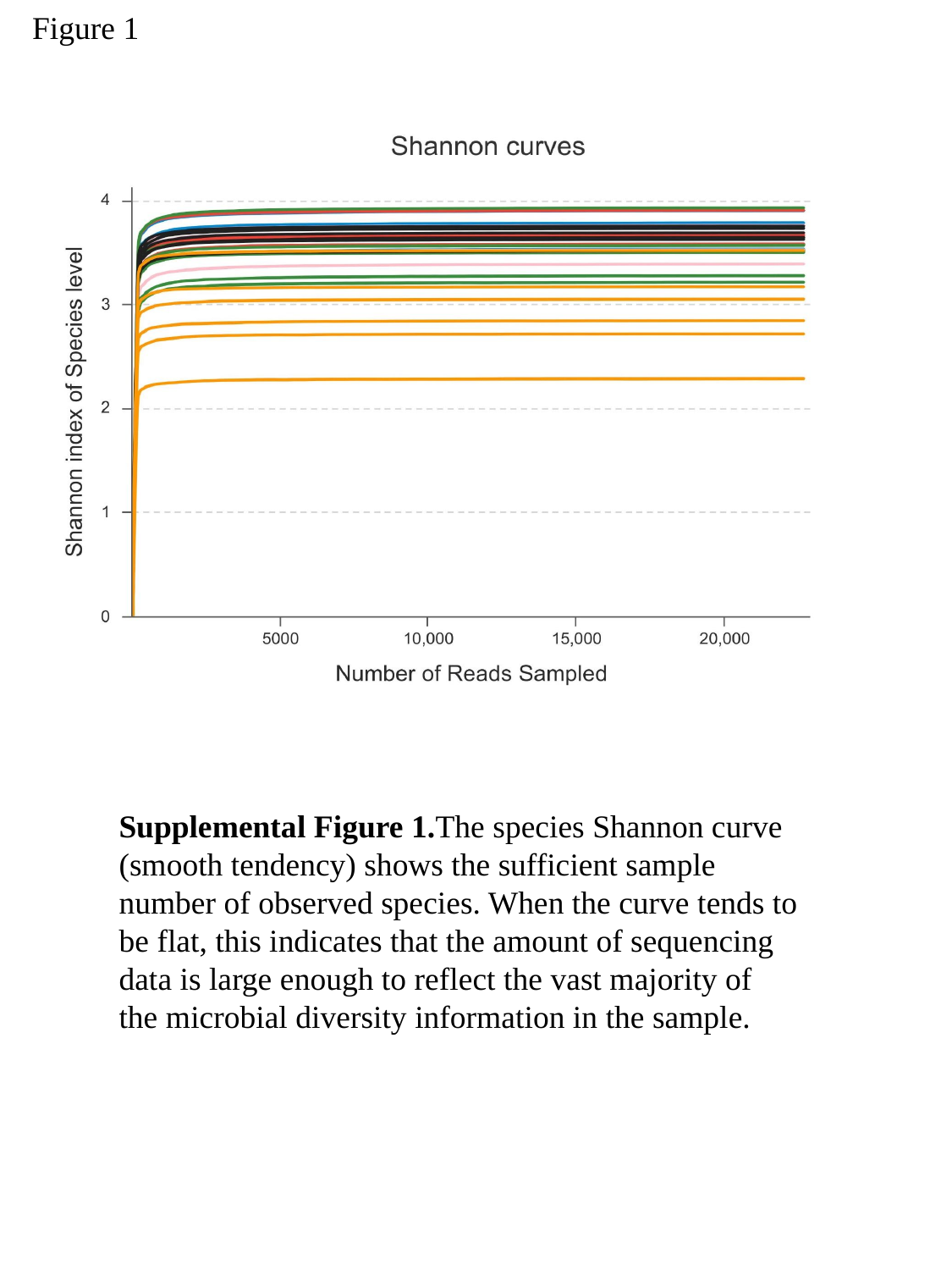

Figure 1
Supplemental Figure 1.The species Shannon curve (smooth tendency) shows the sufficient sample number of observed species. When the curve tends to be flat, this indicates that the amount of sequencing data is large enough to reflect the vast majority of the microbial diversity information in the sample.

## Slide 2
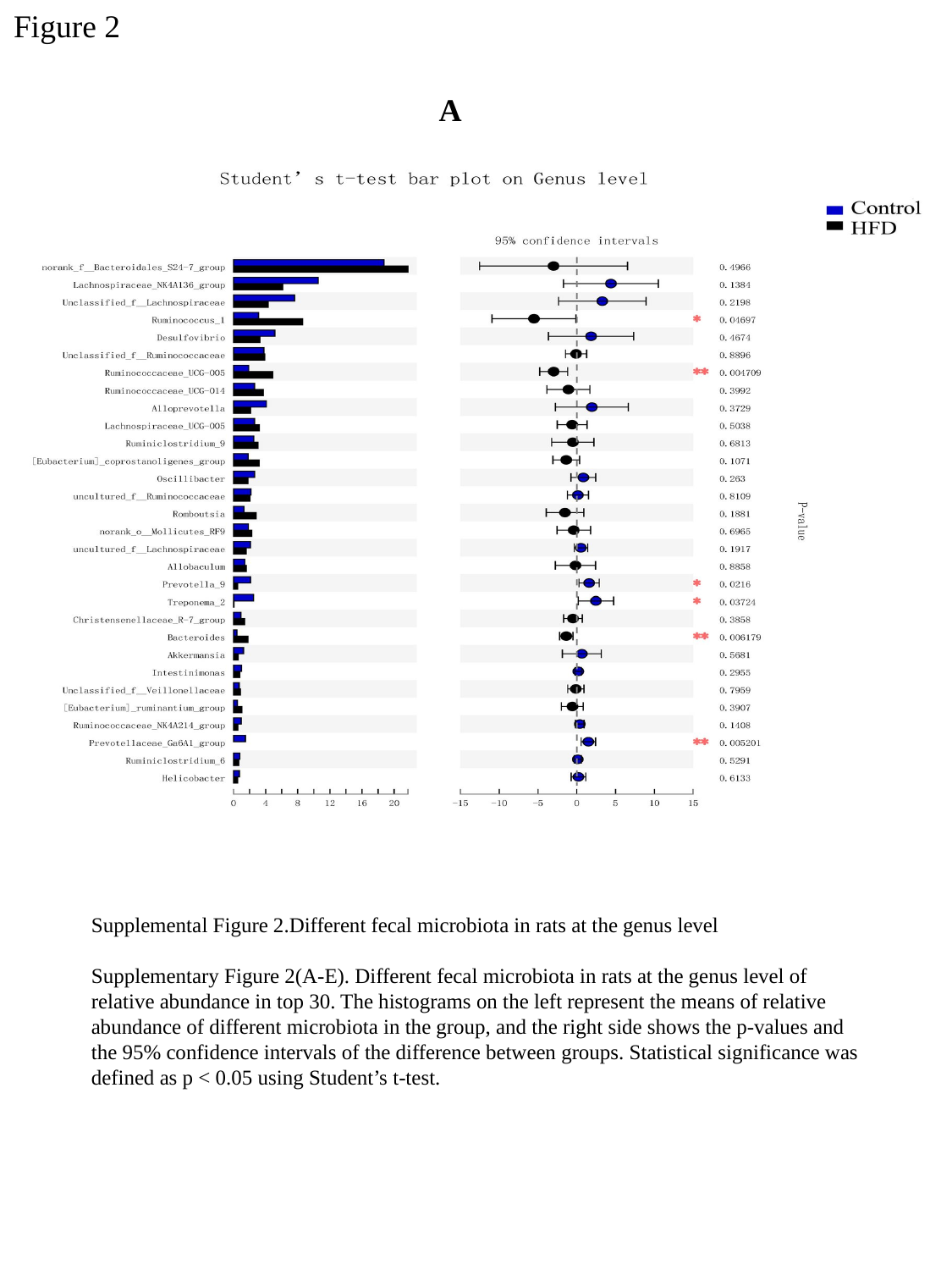

Figure 2
A
Supplemental Figure 2.Different fecal microbiota in rats at the genus level
Supplementary Figure 2(A-E). Different fecal microbiota in rats at the genus level of relative abundance in top 30. The histograms on the left represent the means of relative abundance of different microbiota in the group, and the right side shows the p-values and the 95% confidence intervals of the difference between groups. Statistical significance was defined as p < 0.05 using Student’s t-test.

## Slide 3
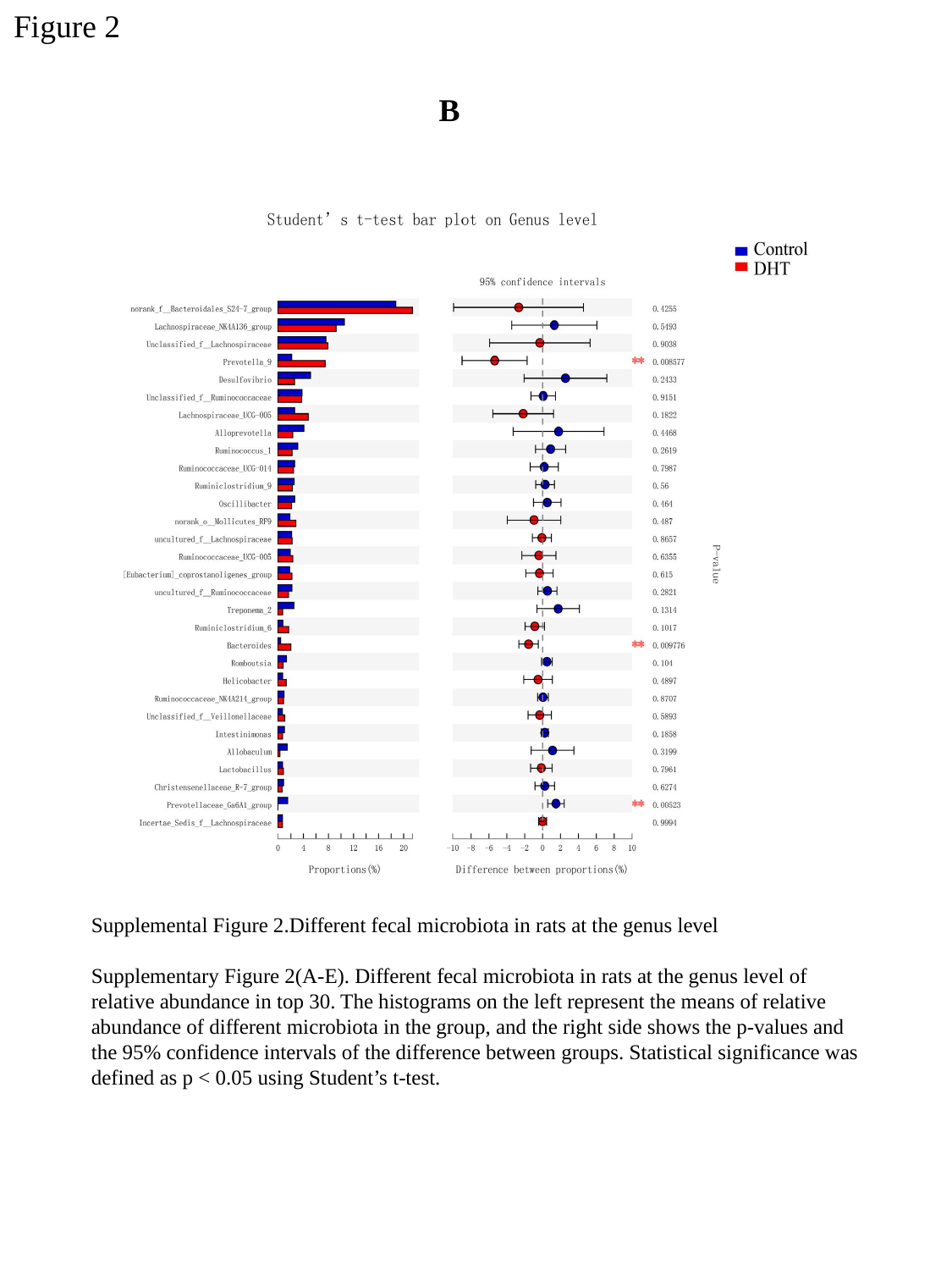

Figure 2
B
Supplemental Figure 2.Different fecal microbiota in rats at the genus level
Supplementary Figure 2(A-E). Different fecal microbiota in rats at the genus level of relative abundance in top 30. The histograms on the left represent the means of relative abundance of different microbiota in the group, and the right side shows the p-values and the 95% confidence intervals of the difference between groups. Statistical significance was defined as p < 0.05 using Student’s t-test.

## Slide 4
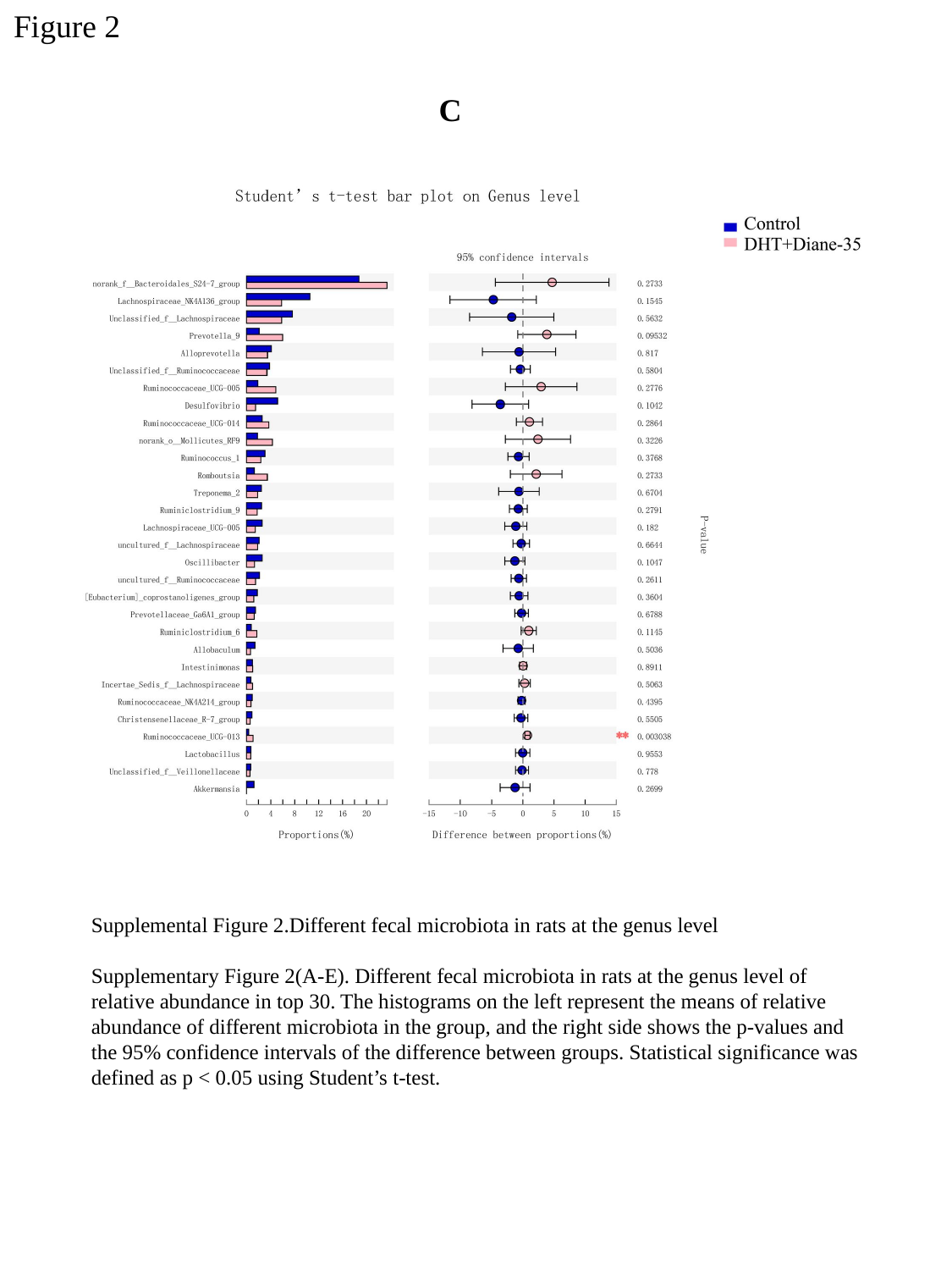

Figure 2
C
Supplemental Figure 2.Different fecal microbiota in rats at the genus level
Supplementary Figure 2(A-E). Different fecal microbiota in rats at the genus level of relative abundance in top 30. The histograms on the left represent the means of relative abundance of different microbiota in the group, and the right side shows the p-values and the 95% confidence intervals of the difference between groups. Statistical significance was defined as p < 0.05 using Student’s t-test.

## Slide 5
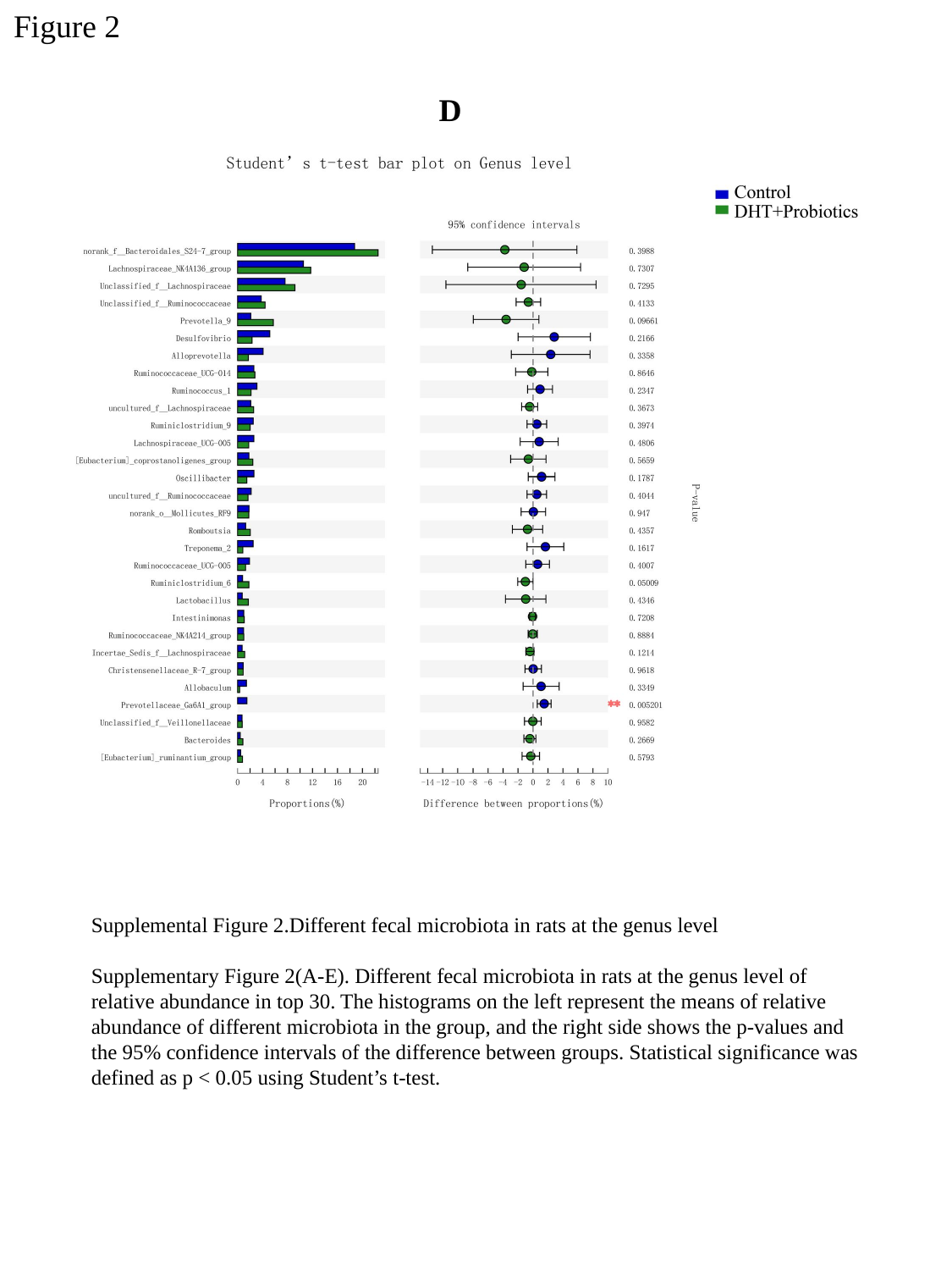

Figure 2
D
Supplemental Figure 2.Different fecal microbiota in rats at the genus level
Supplementary Figure 2(A-E). Different fecal microbiota in rats at the genus level of relative abundance in top 30. The histograms on the left represent the means of relative abundance of different microbiota in the group, and the right side shows the p-values and the 95% confidence intervals of the difference between groups. Statistical significance was defined as p < 0.05 using Student’s t-test.

## Slide 6
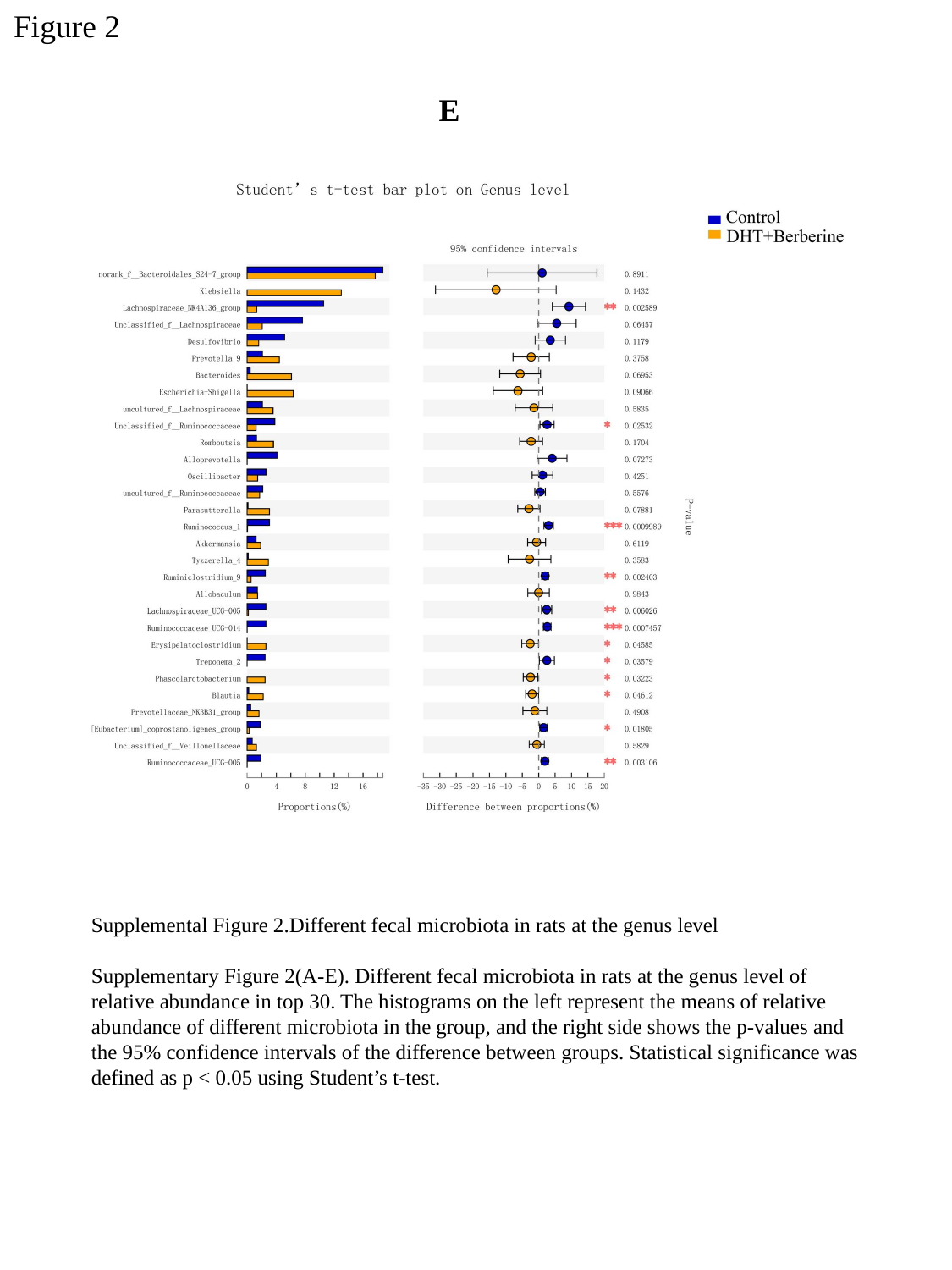

Figure 2
E
Supplemental Figure 2.Different fecal microbiota in rats at the genus level
Supplementary Figure 2(A-E). Different fecal microbiota in rats at the genus level of relative abundance in top 30. The histograms on the left represent the means of relative abundance of different microbiota in the group, and the right side shows the p-values and the 95% confidence intervals of the difference between groups. Statistical significance was defined as p < 0.05 using Student’s t-test.
